# Microwave-Assisted γ-Valerolactone Production for Biomass Lignin Extraction: A Cascade Protocol

**DOI:** 10.3390/molecules21040413

**Published:** 2016-03-26

**Authors:** Silvia Tabasso, Giorgio Grillo, Diego Carnaroglio, Emanuela Calcio Gaudino, Giancarlo Cravotto

**Affiliations:** 1 Dipartimento di Chimica, University of Turin, Via P. Giuria 7, 10125 Turin, Italy; silvia.tabasso@unito.it; 2 Dipartimento di Scienza e Tecnologia del Farmaco and NIS-Centre for Nanostructured Interfaces and Surfaces, University of Turin, Via P. Giuria 9, 10125 Turin, Italy; giorgio.grillo@unito.it (G.G.); d.carnaroglio@milestonesrl.com (D.C.); emanuela.calcio@unito.it (E.C.G.); 3 Milestone Srl, Via Fatebenefratelli 1/5, 24010 Sorisole (BG), Italy

**Keywords:** lignin, biomass, γ-valerolactone, microwaves, bio-refinery

## Abstract

The general need to slow the depletion of fossil resources and reduce carbon footprints has led to tremendous effort being invested in creating “greener” industrial processes and developing alternative means to produce fuels and synthesize platform chemicals. This work aims to design a microwave-assisted cascade process for a full biomass valorisation cycle. GVL (γ-valerolactone), a renewable green solvent, has been used in aqueous acidic solution to achieve complete biomass lignin extraction. After lignin precipitation, the levulinic acid (LA)-rich organic fraction was hydrogenated, which regenerated the starting solvent for further biomass delignification. This process does not requires a purification step because GVL plays the dual role of solvent and product, while the reagent (LA) is a product of biomass delignification. In summary, this bio-refinery approach to lignin extraction is a cascade protocol in which the solvent loss is integrated into the conversion cycle, leading to simplified methods for biomass valorisation.

## 1. Introduction

A number of protocols which convert lignocellulosic biomass to value-added platform molecules and biofuels have been described over the last decade. γ-Valerolactone (GVL) is the most attractive and versatile lignocellulose chemical derivative as it is directly used as a fragrance, liquid fuel and food additive [1,2,3,4]. Furthermore, it is an intermediate in the production of fuel additives, biomass-derived polymers, short chain alkenes (jet fuel) and long chain alkanes (diesel fuel) [5,6,7]. GVL is renewable, safe to store, biodegradable and can be used as a green solvent in fine chemicals synthesis [8] and biomass processing [5]. In fact, GVL is commonly synthesized from levulinic acid (LA) via hydrogenation to 4-hydroxypentanoic acid, which spontaneously condensates to GVL [9]. Several procedures have been carried out under homogeneous and heterogeneous [10] conditions for selective LA hydrogenation [11]. Most processes use molecular H_2_ as the reducing agent, supported metals as catalysts (Ir, Rh, Pd, Ru, Pt, Re and Ni) and dioxane as the solvent [5]. The highest GVL yields have either been obtained under severe reaction conditions (200 °C and 200 bar H_2_), in the presence of a co-catalyst (Amberlyst A70 or A15) or an active hydrogen-donor (formic acid, FA) [12]. Recent studies on “greener” methods have moved on from the use of alcohols and ethers as solvents [13,14] to LA hydrogenation in water and milder conditions [15]. Although only a small number of investigations into the solvent-free conversion of LA into GVL have so far been published, a great deal of work to enhance the sustainability of this reaction is still in progress. Ni/Al_2_O_3_ catalysts have been tested in batch autoclaves [16], in quite harsh, but solventless, conditions.

It has recently been demonstrated that GVL can completely solubilise the biomass, thus promoting the production of sugars through thermocatalytic saccharification [17]. Wettstein *et al.* have reported the use of GVL as a solvent in the production of LA and GVL from cellulose [18], and, alternatively, from lignocellulosic biomass [19]. These studies focused on the sugar-derived products, without any further lignin processing, although it is considered the main renewable source of aromatics. Lignin extraction from lignocellulosic biomass (wood, annual plants) is the key step in large scale use for industrial applications. Lignin from the pulp and paper industries is generally only used as fuel to power paper mills and is no longer considered waste. It has, however, attracted increasing interest over the last few years as a means to produce dispersants, adhesives and surfactants, as an antioxidant in plastics and rubbers, as well as the raw material for the synthesis of value-added products [20]. One common method for the extraction of lignin draws on solvent pulping (organosolv lignin). The structure of these lignins is close to those of the native lignins [21,22]. Most common organosolv processes are based on ethanol/water or acetic acid containing a small amount of mineral acid such as hydrochloric or sulfuric acid [23]. A dioxasolv process has recently been used to extract lignin from birch (*Betula pendula*) sawdust, using a diluted hydrochloric acid solution in dioxane [24]. This process leads to high extraction yields but involve several extraction/precipitation steps with organic solvents.

The last three decades have seen several attempts to improve the sustainability of a variety of chemical processes being performed in non-conventional enabling technologies, such as microwaves (MW) [25] and ultrasound (US) reactors [26]. The peculiar physical properties of MW may significantly promote biopolymers hydrolysis and their further conversion into platform chemicals [27]. Cellulose and lignocellulosic biomass can be converted to LA under MW-assisted protocols and provide full conversion and good selectivity [28,29]. The hydrogenation of LA under MW irradiation has recently been studied with FA being employed as a hydrogen-donor solvent [30]. In this process, 2-methyltetrahydrofuran (MTHF) was produced, with some by-products, in 30 min at 150 °C.

The present work reports a cascade MW-assisted integrated protocol which exploits the biorefinery approach to “green” hydrogenation of biomass-derived LA to GVL, and its recycling for further lignin extraction from biomass.

## 2. Results and Discussion

### 2.1. Synthesis of GVL from LA

**Scheme 1:**
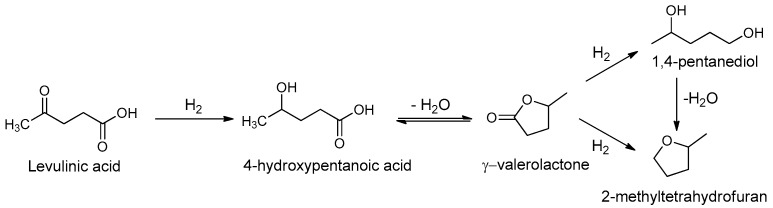
Reaction pathway for the production of GVL and further hydrogenation products from LA.

One pathway to GVL relies on the hydrogenation of LA to 4-hydroxypentanoic acid; an unstable intermediate which undergoes intramolecular esterification with loss of a water molecule to afford GVL [31]. The highest yields were achieved using Ru-based catalysts [10]. Other noble metals, such as Pt and Pd, also catalysed GVL hydrogenation to afford 2-methyltetrahydrofuran (MTHF) and 1,4-pentanediol in spite of high GVL production rates (Scheme 1) [11,32].

Bermudez *et al.* reported the hydrogenation of LA over Pd/C under MW irradiation with FA as the hydrogen source, however the reaction proceeded to MTHF with a LA/catalyst ratio of 1/1 (mL/g) [30]. Our recent findings on the flash MW conversion of biomass into LA provided a base for our experiments into a MW-assisted synthesis of GVL using economical and commercially available Pd/C as catalyst and molecular H_2_ as the reducing agent. The main target of the present study was to perform a “green” hydrogenation in water or in solvent-free conditions and thus improve the sustainability of the chemical process (Table 1). When the reaction was carried out in water, a significant amount of 4-hydroxypentanoic acid was found (Table 1, entry 1), because of an equilibrium shift in the intramolecular esterification. Contrary to data published by Tan *et al.* on hydrogenation in water [33], our experiments found that water disfavors intramolecular esterification. We therefore studied the reaction in solvent-free conditions, varying the reaction temperature, the amount of catalyst and hydrogen pressure. The complete conversion and good selectivity were obtained using 50 bar H_2_ for 4 h at 150 °C (Table 1, entries 3 and 4). Most research on LA hydrogenation to GVL has made use of pure, commercial LA and processing conditions that are not suitable for use in cascade processes that operate with streams of lignocellulosic biomass derived LA. The solvent-free protocol shed light on water’s role in the hydrogenation process, but could not be used in the cascade process where the GVL plays a pivotal role. For this reason, we performed the hydrogenation of biomass-derived LA, using GVL as a solvent, under the optimized conditions (Table 1, entry 7). The LA obtained from the biomass conversion, carried out according to our previous work [29], afforded GVL with complete conversion and selectivity. No further hydrogenation products were detected. Product separation and purification were not required when using GVL as the solvent.

### 2.2. Extraction of Lignin from Biomass

The use of GVL/H_2_O mixtures for the fractionation of lignocellulosic biomass in a MW reactor at 170 °C for 2 h has recently been reported. Acidic catalysis (H_2_SO_4_) yielded pure cellulose and a pure lignin fraction [34]. For our own aims, an integrated, MW-assisted flash biomass delignification process (2 min) was carried out using an acidic aqueous system GVL/H_2_O = 1:1 (HCl, 1M). The biomass was made up of post harvested tomato plants, whose composition was investigated in a previously described procedure (Table 2) [35].

**Figure 1:**
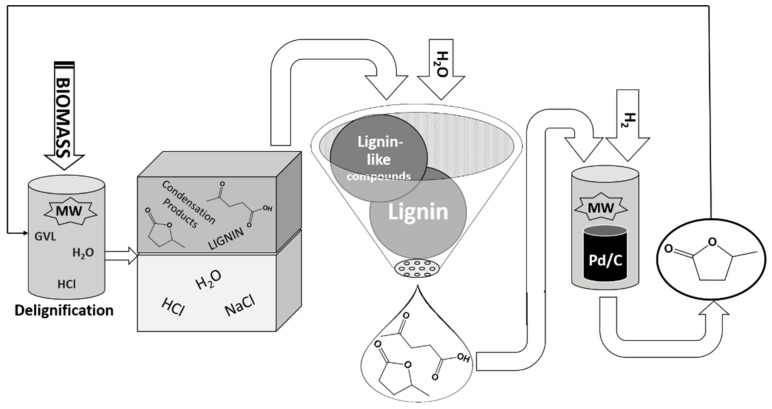
Scheme of the cascade process for the extraction of lignin.

The cellulose fraction was rapidly converted to LA [29], and then subsequently hydrogenated to regenerate and recycle the solvent in a cascade process (Figure 1) where the lignin was extracted via simple precipitation. GVL is stable in acidic systems at high temperatures (225 °C) [17], and the GVL/H_2_O mixtures facilitate the complete solubilisation of biomass (99.10%). As GVL solvent leads to higher rates of acid-catalyzed reactions than to reactions in water [36], hemicellulose was immediately hydrolyzed to oligomeric and monomeric xylose and the dehydration product furfural. Sugars that are derived from the hydrolysis of cellulose and hemicellulose, and especially furfural, can further react to lignin-like condensation products, which are soluble in the solvent mixture. The addition of NaCl after the MW-assisted process favored the separation of the GVL layer from the HCl water solution.

In the biphasic system, most of the LA was partitioned in the organic phase (93% of the total LA obtained at the end of the process). Thus, after separation, the organic phase contained GVL, LA, lignin and the lignin-like condensation products, which were eventually formed by cellulose and hemicellulose degradation. Lignin and the lignin-like condensation products, in particular, were recovered by precipitation after dilution, of over 10 times, with distilled water [18]. This procedure yielded a 19 wt % precipitate, compared to the starting biomass, while the lignin in the raw biomass was 15 wt %. This excess comes from the formation of a few lignin-like degradation products. 

**Figure 2:**
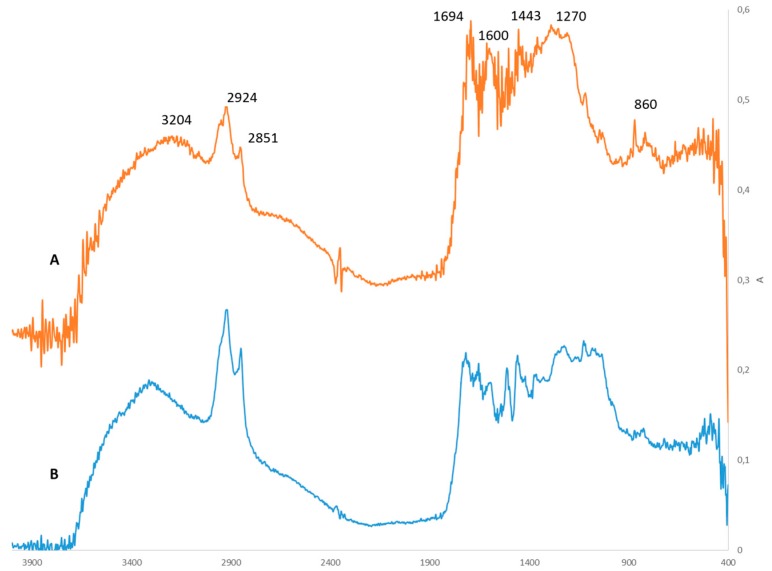
IR spectra of lignin obtained from biomass deconstruction in GVL/H_2_O (**A**) and dioxasolv lignin (**B**).

IR spectra of the precipitate (Figure 2A) were very similar to those of the lignin extracted using a dioxasolv process (Figure 2B). Characteristic lignin bands for O–H stretching (3204 cm^−1^ respectively), C-H stretching (2924 and 2851 cm^−1^), and aromatic skeletal vibrations (1600 cm^−1^) were observed. The band at 1694 cm^−1^ belongs to the stretching of the C=O functional groups. The CH_2_ deformation of aliphatic chains is also present (1443 cm^−1^). The broad band at 1270 cm^−1^ belongs to the guaiacyl unit, as does that at 860 cm^−1^.

The remarkable features of this method are: (i) a one-step flash-treatment for both the solubilisation of the lignin and the conversion of cellulose to LA; (ii) the absence of a purification step for the recovery of LA; (iii) the dual role of GVL as the solvent and product to be recycled. This process complies with the six principles of green extraction [37,38]. In particular, alternative bio-based solvents have been used to extract lignin from a renewable source with an integrated approach by innovative and energy-saving technologies.

### 2.3. Regeneration of the Solvent

**Figure 3:**
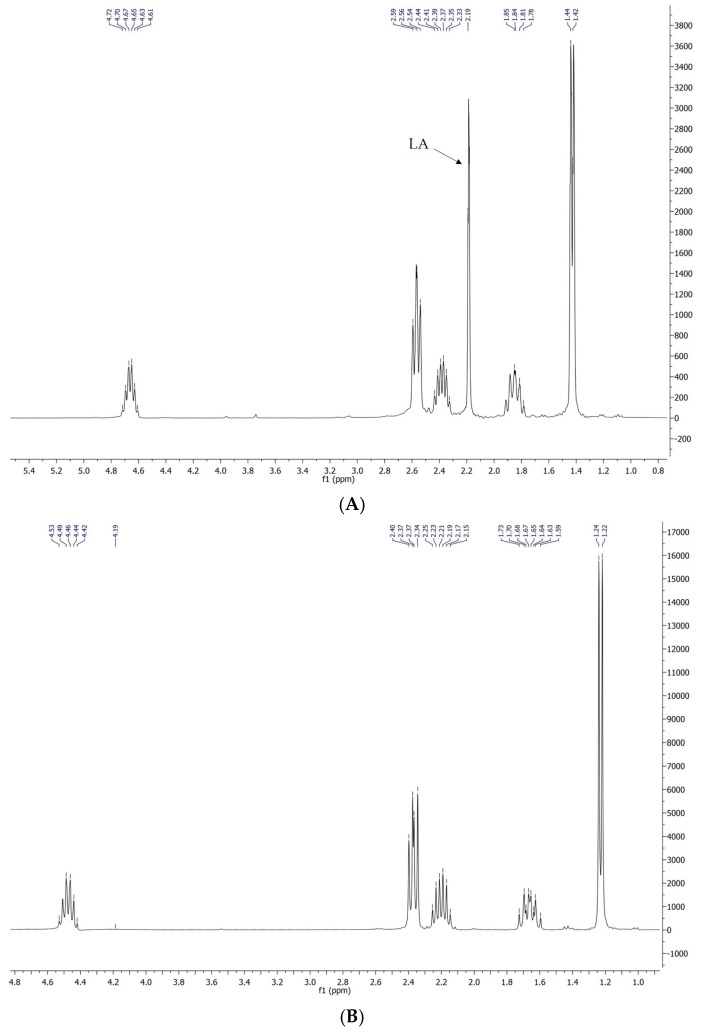

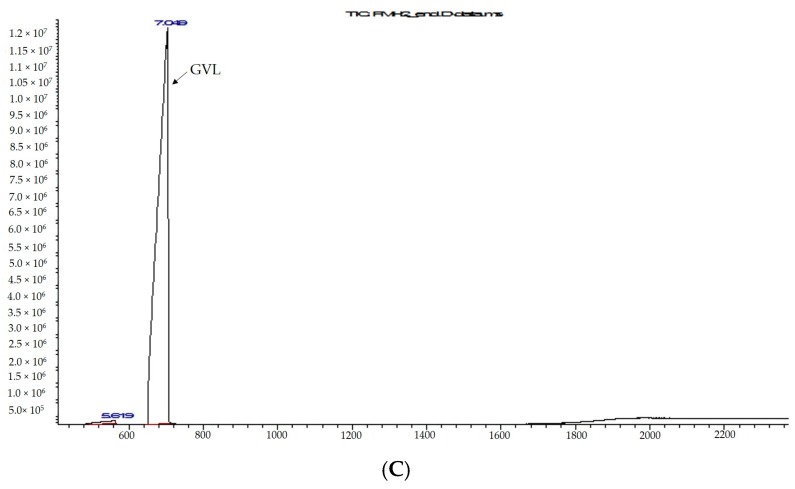
(**A**) ^1^H-NMR of the organic phase after the precipitation of lignin; (**B**) ^1^H-NMR of the same solution after hydrogenation; (**C**) GC-MS of the same solution after hydrogenation.

The organic phase that remained after the precipitation of lignin contained only LA and GVL, as confirmed by GC-MS and ^1^H-NMR (Figure 3A). It was thus used as the feedstock for the regeneration of the solvent. The hydrogenation step was performed using Pd/C as the catalyst, under the optimized conditions reported above for the reduction of biomass-derived LA in GVL (Table 1, entry 7).

Researchers that used biomass derived LA have observed that mineral acids present in the feed deactivate the catalyst [18]. In our case, no deactivation was observed and LA was fully converted to GVL. As shown in Figure 3, the ^1^H-NMR signal at 2.19 ppm, which corresponds to the LA methyl group, disappeared after hydrogenation, as confirmed both by NMR (Figure 3B) and GC-MS (Figure 3C), where the only peak detected belongs to GVL. This demonstrates the efficiency of a separation method that ensured the complete washing of HCl in water during the first separation step.

In summary, we report the first example of a cascade process where a real organic feed obtained after biomass deconstruction was applied to regenerate the solvent for the process. Moreover, the MW-assisted flash conversion was able to synergistically improve the efficiency and sustainability of biomass valorisation process.

## 3. Experimental Section

### 3.1. General Information

All chemicals and solvents were purchased from Sigma-Aldrich (St. Louis, MO, USA)) and used without further purification. Post-harvest tomato plants were blade milled. MW-assisted reactions were carried out in a high-pressure professional MW reactor (200 bar), SynthWave (Milestone Srl, MLS GmbH, Bergamo, Italy). NMR spectra were recorded on a Avance 300 (300 MHz) spectrometer (Bruker, Madison, WI, USA), at 25 °C; chemical shifts were calibrated to the residual solvent proton. GC-MS analyses were performed in a GC Agilent 6890 (Agilent Technologies, Santa Clara, CA USA) which was fitted with an Agilent Network 5973 mass detector, using a HP-5 column (30 m long capillary column, i.d of 0.25 mm and film thickness 0.25 μm). Infrared spectra (FTIR) were recorded using a KBr powder and a Spectrum BX FT-IR System (Perkin Elmer, Waltham, MA, USA).

### 3.2. Synthesis of GVL from LA and Analysis

In a typical experiment, the selected volume of LA was directly added to the Pd/C catalyst (10 wt % loading), followed by distilled water or GVL where needed. The required temperature was rapidly achieved in the MW reactor (average power 1500 W), under H_2_ pressures and magnetic stirring (600 rpm). After cooling, the solution was filtered and the solid was washed three times with CHCl_3_ (3 × 5 mL).

After solvent evaporation under vacuum, the crude reaction was analyzed by ^1^H-NMR (CDCl_3_) and GC-MS. GC-MS chromatograms were recorded after preliminary dilution with chloroform (1:50 for 0.1 mL LA and 1:1000 for 4 mL LA) and derivatization. In a typical procedure, 40 μL *N*,*O*-*bis*(trimethylsilyl)trifluoroacetamide (BSTFA) were added to 2 mL of product solution. The mixture was heated for 45 min. at 60 °C under magnetic stirring and the product was directly analysed. GC conditions were: injection split 1:20, injector temperature 250 °C, detector temperature 280 °C. Gas carrier: helium (1.2 mL/min), temperature program: From 70 °C (2 min) to 300 °C at 5 °C/min.

### 3.3. Extraction of Lignin from Biomass and Analysis

The biomass (1 g) was suspended in a GVL/acidic aqueous solution (1:1, 1M HCl, total volume 20 mL). The mixture was irradiated in a MW reactor under N_2_ at 225 °C (average power 1500 W) with magnetic stirring (600 rpm). The crude reaction was filtered under vacuum after 2 min. The dried solid was weighed (wt % of solubilisation). The liquid fraction was separated into two phases by adding a saturated NaCl solution (30 mL). The organic fraction was then treated with 100 mL water which provided the lignin and lignin-like precipitation. The mixture was filtered and water removed under vacuum. The final solution was analyzed by ^1^H-NMR (CDCl_3_) and GC-MS, after prior derivatization of a diluted sample (1:10) in chloroform with BSTFA (as reported above). All the solid residues were accurately washed with a known volume of pure GVL/water solution (three times, 4 mL). The filter was washed with a large amount of distilled water after filtration, assuring the complete elimination of the organic solvent. The precipitate was dried in an oven at 30 °C overnight and then for two hours under vacuum.

### 3.4. Regeneration of the Solvent and Analysis

The solution recovered after lignin precipitation was hydrogenated under MW irradiation without any further purification or separation step, according to the optimized procedure for biomass-derived LA. The catalyst excess was calculated for the complete conversion of cellulose. After 4 h, the crude reaction was cooled down, filtered, and the solution analyzed using ^1^H-NMR (CDCl_3_) and GC-MS after prior derivatization.

## 4. Conclusions

A cascade MW-assisted integrated process has been developed for the extraction of lignin from biomass by mean of a “green” and renewable solvent produced and regenerated in the same process. The acidic aqueous GVL solution afforded the complete solubilisation of the biomass deconstruction products and the facile recovery of lignin by simple precipitation. In addition, the MW-assisted hydrogenation of the obtained LA to GVL eliminates the need for a separation step, as GVL is both solvent and product. The lignin extracted from the biomass was very similar to samples obtained using other extraction methods involving organic solvents and numerous steps. This method for lignin extraction may well lead to an upgrade in the subsequent lignin valorisation processes.

## Figures and Tables

**Table 1:** MW-assisted hydrogenation of LA ^a^.

Entry	LA (mol)	Pd/C (wt %)	Solvent	H_2_ (bar)	Temperature (°C)	LA Conversion ^b^ (%)	GVL Selectivity ^b^ (%)
1	1	35	H_2_O	50	150	100	10 ^c^
2	1	35	-	100	90	100	100
3	1	35	-	50	150	100	100
4	40	10	-	50	150	100	100
5	40	10	-	50	90	39	100
6	40	4	-	50	150	86	100
7 ^d^	10	10	GVL ^e^	50	150	100	100

^a^ Reaction time: 4 h; ^b^ Determined by GC-MS; ^c^ 4-hydroxypentanoic acid was the major product; ^d^ Biomass-derived LA; ^e^ LA/GVL = 1/2.

**Table 2:** Composition of biomass (wt %).

Post-Harvested Tomato Plants	
Ash	22%
Proteins and lipids	3%
Cellulose	39%
Hemicellulose	21%
Lignin	15%
